# Non-cirrhotic portal-systemic encephalopathy caused by enlargement of a splenorenal shunt after pancreaticoduodenectomy for locally advanced duodenal cancer: report of a case

**DOI:** 10.1007/s00595-013-0679-1

**Published:** 2013-08-28

**Authors:** Yuta Kasagi, Hiroshi Saeki, Tomohiko Akahoshi, Junji Kawasaki, Koji Ando, Eiji Oki, Takefumi Ohga, Morimasa Tomikawa, Yoshihiro Kakeji, Ken Shirabe, Yoshihiko Maehara

**Affiliations:** Department of Surgery and Science, Graduate School of Medical Sciences, Kyushu University, Fukuoka, 812-8582 Japan

**Keywords:** Encephalopathy, Splenorenal shunt, Duodenal cancer, Pancreaticoduodenectomy, Balloon-occluded retrograde transvenous obliteration

## Abstract

We report a case of portal-systemic encephalopathy occurring secondary to a splenorenal shunt, 2 years after a pancreaticoduodenectomy for locally advanced duodenal carcinoma. A 55-year-old woman was brought to our hospital with a decreased level of consciousness. Laboratory testing revealed an elevated serum ammonia level (221 μg/dl) and normal liver function. Retrospective review of a series of contrast-enhanced computed tomography scans of the abdomen identified a splenorenal shunt, which had gradually enlarged over the past 2 years (Fig. 1). The decreased level of consciousness was thought to be due to portal-systemic encephalopathy secondary to the splenorenal shunt. We performed balloon-occluded retrograde transvenous obliteration to occlude the splenorenal shunt, following which her serum ammonia level returned to normal (28 μg/dl) and an alert level of consciousness was maintained.

**Fig. 1:**
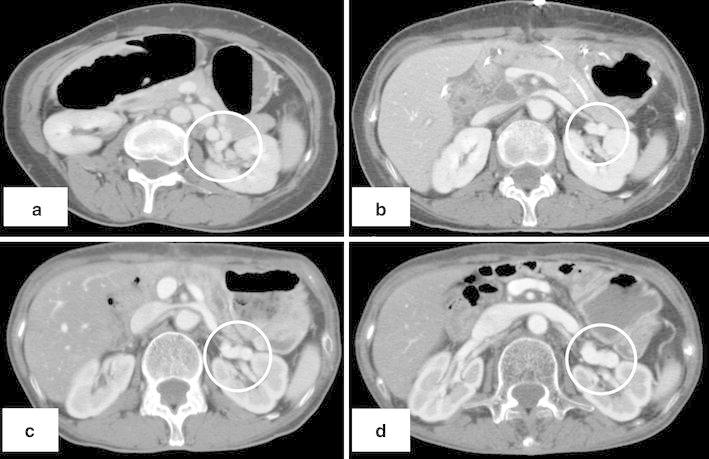
Review of abdominal computed tomography scans. **a** Preoperatively, **b** 6 months postoperatively, **c** 1 year postoperatively, **d** 2 years and 2 months postoperatively. The shunt vessel gradually enlarged after pancreaticoduodenectomy (*circle*)

Review of abdominal computed tomography scans. **a** Preoperatively, **b** 6 months postoperatively, **c** 1 year postoperatively, **d** 2 years and 2 months postoperatively. The shunt vessel gradually enlarged after pancreaticoduodenectomy (*circle*)

## Introduction

A portal-systemic shunt may cause fluctuating levels of consciousness and abnormal behavior, because of the diversion of portal blood directly into the venous circulation [1]. This condition typically occurs secondary to liver cirrhosis or idiopathic portal hypertension; however, it may also occur in patients with normal liver function and normal portal pressure, in which case it is called “non-cirrhotic portal-systemic encephalopathy” [2]. We report an unusual case of non-cirrhotic portal-systemic encephalopathy caused by a splenorenal shunt that enlarged after pancreaticoduodenectomy for locally advanced duodenal cancer. The patient was successfully treated by balloon-occluded retrograde transvenous obliteration (B-RTO).

## Case report

**Fig. 2:**
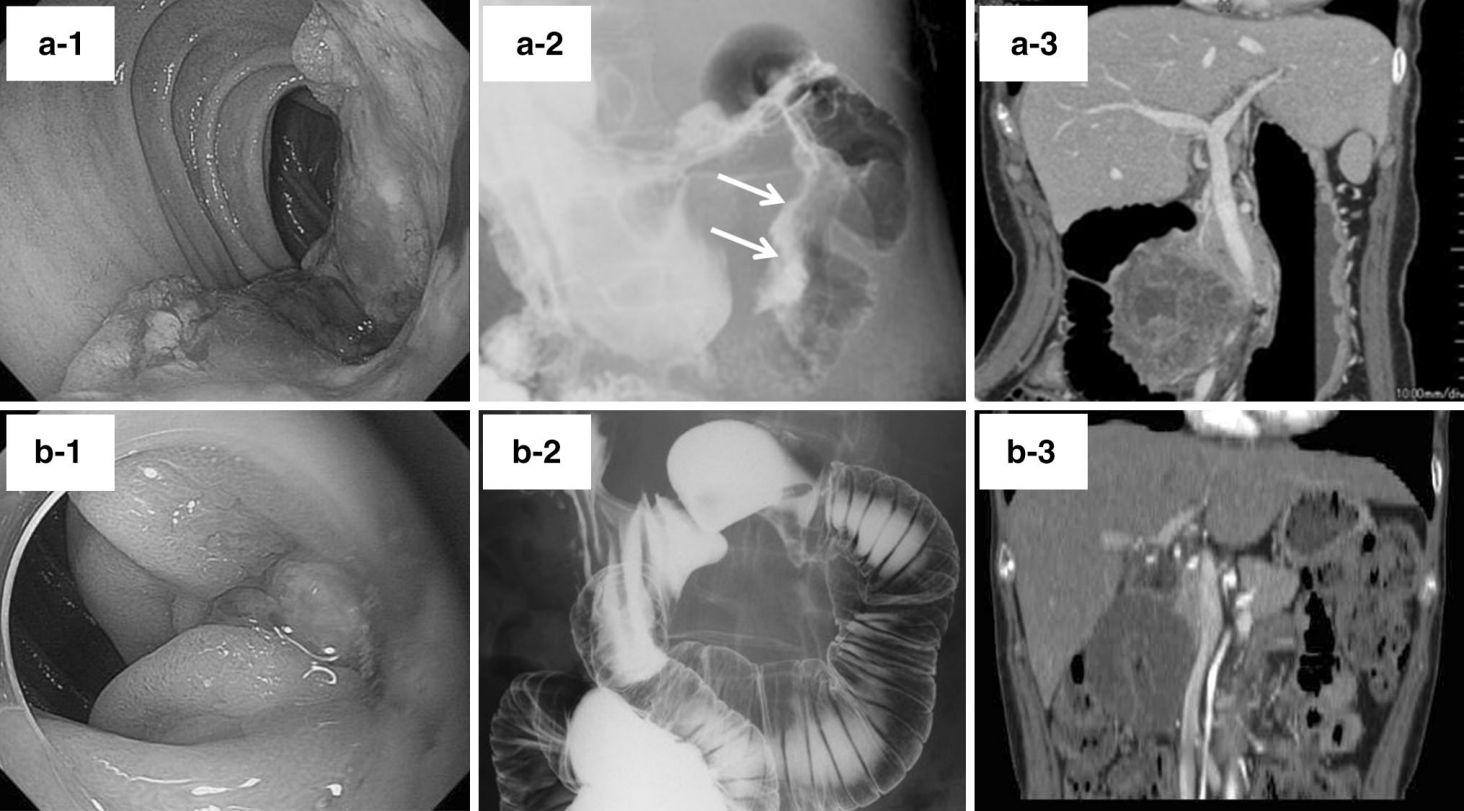
Imaging examinations. **a** Before chemotherapy. **b** After chemotherapy. **a-1** Upper gastrointestinal endoscopy showing Borrmann’s classification type 2 cancer in the second part of the duodenum. **a-2** Hypotonic duodenography showing the tumor (*arrow*). **a-3** Abdominal contrast-enhanced computed tomography, showing a tumor 9 cm in diameter with invasion of the pancreatic head and superior mesenteric vein (SMV). **b1-3** After chemotherapy, the tumor had decreased in size and invasion of the SMV had regressed

A 55-year-old woman was brought to our hospital by ambulance because of a reduced level of consciousness. She had undergone pancreaticoduodenectomy for locally advanced duodenal carcinoma more than 2 years earlier, after receiving neoadjuvant chemotherapy with cisplatin and S-1 [3]. Imaging studies before the chemotherapy had revealed Borrmann’s classification type 2 cancer in the second part of the duodenum, with invasion of the pancreatic head and superior mesenteric vein (SMV; Fig. 2a). After the neoadjuvant chemotherapy, the tumor diameter had decreased and invasion of the SMV had regressed (Fig. 2b). No signs of liver cirrhosis were observed intraoperatively, the SMV and inferior mesenteric vein (IMV) were preserved, and the digestive tract was reconstructed using a modification Child’s method. She received postoperative adjuvant chemotherapy with oral S-1. She required several subsequent hospitalizations for malnutrition, but remained free of recurrence or metastasis.

On admission, she had a Japan Coma Scale score of I-2, a heart rate of 120 beats/min, and a blood pressure of 88/55 mmHg. Her body mass index was 13.5 kg/m^2^. Laboratory testing showed a serum ammonia level of 221 μg/dl, a serum albumin level of 1.9 g/dl, an aspartate aminotransferase level of 53U/l, an alanine aminotransferase level of 41U/l, and a prothrombin time (PT) of 57 %, indicating hyperammonemia without severe liver dysfunction. Retrospective review of a series of contrast-enhanced computed tomography (CT) scans of the abdomen identified a splenorenal shunt that had gradually enlarged over the past 2 years (Fig. 1). There was no radiological evidence of cirrhotic change in the liver. Head CT and magnetic resonance imaging revealed no abnormalities of the central nervous system. Thus, her reduced level of consciousness was attributed to hyperammonemia caused by the splenorenal shunt.

**Fig. 3:**
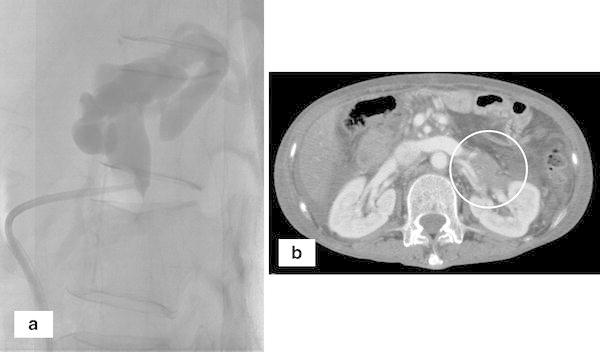
Balloon-occluded retrograde transvenous obliteration. **a** Before the procedure, there was significant dilation of the splenorenal shunt. **b** The injection of 20 ml of 5 % ethanolamine oleate iopamidol eliminated flow through the shunt, and a follow-up CT scan on day 3 showed good occlusion of the shunt (*circle*)

The patient was treated with an intravenous infusion of branched-chain amino acid solution and oral lactulose, following which her level of consciousness improved and her serum ammonia level normalized. The shunt was occluded by B-RTO on the 13th day after admission. A balloon catheter (6.5 French; Create Medic, Tokyo, Japan) was inserted via the right femoral vein and inferior vena cava, and then advanced to the left renal vein and the splenorenal shunt. The shunt was visualized using iopamidol. Inflation of the balloon blocked flow through the shunt (Fig. 3a). The hepatic venous pressure gradient (HVPG) was 7 cmH_2_O. The small drainage veins were collapsed by an injection of 50 % glucose (20 ml) and absolute ethanol (3.5 ml). A slow retrograde injection of 5 % ethanolamine oleate iopamidol (20 ml) sufficiently obliterated the shunt and feeding vessel. The balloon was deflated after 24 h and a follow-up CT scan 3 days later showed good occlusion of the splenorenal shunt (Fig. 3b). The serum ammonia level remained within the normal range, and a normal level of consciousness was maintained. She was transferred to another facility after 25 days for rehabilitation.

## Discussion

Non-cirrhotic portal-systemic encephalopathy was first reported by Raskin et al. [4] and its pathological features were described by Inose [5]. In patients with a portal-systemic shunt, neurotoxic substances such as ammonia flow directly into the systemic circulation and may cause encephalopathy without liver cirrhosis [2]. This condition was initially considered rare, but many cases have been reported recently with advances in abdominal imaging techniques. These patients were previously misdiagnosed with dementia, depression, and other psychiatric disorders. Therefore, portal-systemic encephalopathy should be suspected in patients with large portal-systemic shunts and the serum ammonia level checked, followed by initiation of appropriate treatment. Although we did not perform liver biopsy in our patient, her previous operative findings, laboratory test results, and radiological findings suggested normal liver function. Her serum ammonia level had not been measured prior to her presentation with a decreased level of consciousness. Moreover, her malnutrition may have been a result of disorientation caused by her non-cirrhotic portal-systemic encephalopathy. She was almost bedridden during previous admissions for malnutrition, but was able to undertake personal care tasks after undergoing B-RTO.

**Fig. 4:**
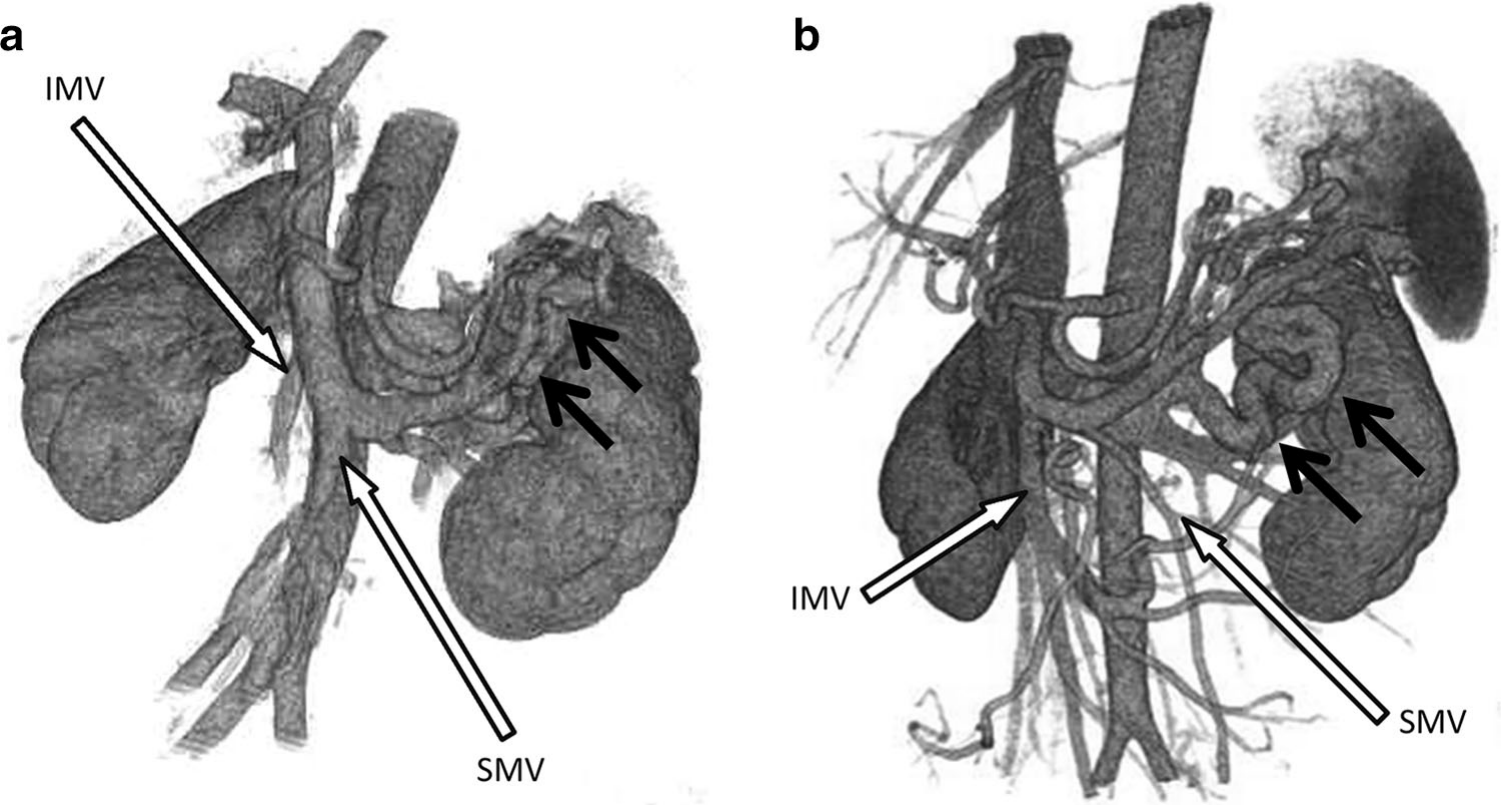
Three-dimensional computed tomography image of the portal venous system. **a** Before tumor resection, the superior mesenteric vein (*SMV*) and inferior mesenteric vein (*IMV*) were very poorly enhanced due to invasion and displacement by the tumor. There was a small splenorenal shunt (*arrow*). **b** After resection, there was substantially increased flow through the SMV and IMV. The IMV became larger than the SMV. The shunt vessel was significantly enlarged (*arrow*)

A non-cirrhotic portal-systemic shunt is generally thought to be due to previous abdominal surgery [6] or a congenital malformation [7]. However, the precise cause is often unclear. A report of 23 cases of portal-systemic encephalopathy indicated that the etiology of shunt formation differed in men and women, being more likely to result from previous surgery in men and from congenital malformations in women [8]. Left-sided portal hypertension is associated with some types of pancreatic neoplasm [9]. The possibility of congenital malformation was not completely ruled out in our patient because there were no preoperative CT images available for comparison. It is also possible that the shunt was caused by left-sided portal hypertension due to the advanced tumor invading the SMV. A retrospective review of three-dimensional CT images of the portal system showed that the SMV and IMV were very poorly enhanced preoperatively because of invasion and displacement by the tumor (Fig. 4a). After the resection, the flow in these veins increased substantially and the IMV became larger than the SMV (Fig. 4b). We speculate that an increase in portal blood flow after surgery caused portal hypertension and subsequent enlargement of the splenorenal shunt, as well as secondary non-cirrhotic portal-systemic encephalopathy.

B-RTO is now generally accepted as the first-line treatment for solitary gastric varices with a gastro-renal shunt in Japan [10, 11]. Reports of treating portal-systemic encephalopathy caused by a splenorenal shunt, by B-RTO, indicate a good prognosis [12, 13]. Our patient underwent B-RTO to close the shunt vessel, with satisfactory results. Therefore, B-RTO seems to be a good treatment choice for patients with non-cirrhotic portal-systemic encephalopathy.

To our knowledge this is the first report of the successful treatment by B-RTO, of non-cirrhotic portal-systemic encephalopathy caused by a gradually enlarging splenorenal shunt after pancreaticoduodenectomy for locally advanced duodenal cancer.
